# Suture Sliding Technique for Arthroscopic Matrix-based Meniscus Repair

**DOI:** 10.1016/j.eats.2025.103520

**Published:** 2025-03-22

**Authors:** Sean Wei Loong Ho, Timothy Zhen Xin Tan, Wei Zhang, T. Jegathesan, Lester Teong Jin Tan, Keng Thiam Lee

**Affiliations:** a Department of Orthopaedic Surgery, Tan Tock Seng Hospital, Singapore; b Yong Loo Lin School of Medicine, National University of Singapore, Singapore

## Abstract

Complex meniscal tears associated with poor meniscal quality can be challenging to repair. A robust repair configuration is required for a successful repair. Blood supply and adequate biology are also important factors to improve healing rates. Biological augmentation of a meniscus repair can be performed via orthobiologics or collagen-based matrices. The aim of this Technical Note is to describe a surgical technique of matrix insertion by sliding the matrix down sutures during arthroscopic matrix-based meniscus repair.

There has been an evolution in the understanding of the role of the meniscus and its protective effect of the knee joint.^1^ Preserving meniscal tissue is now at the forefront of knee preservation, with several consensus statements highlighting the need for meniscal preservation.^1^, ^2^, ^3^ Meniscus tears can be repaired adequately via several surgical techniques, which include the inside-out, outside-in, or all-inside techniques. There are several factors that affect the success rates of meniscus repair, such as chronicity of injury, location of meniscus tear, geometry of meniscus tear, and quality of meniscal tissue.^4^, ^5^, ^6^, ^7^ In particular, the presence of adequate vascularity and biology is an important factor that determines meniscus healing.^4^, ^8^, ^9^, ^10^ Meniscus trephination and notch microfracture are some techniques employed to improve vascularity.^8^, ^9^, ^10^ To further improve the biological environment, biologic adjuncts such as platelet-rich plasma, bone marrow aspirate concentrate (BMAC), and collagen-based matrices can be used.^8^, ^9^, ^10^, ^11^, ^12^, ^13^, ^14^, ^15^, ^16^ One technique used to incorporate these biological augments is the arthroscopic matrix-based meniscus repair. This technique was originally described by Piontek et al.^12^ in 2012 and consists of meniscal repair with a collagen scaffold accompanied by bone marrow blood aspirate injection.

Although this technique is relatively straightforward in approach, insertion and appropriate placement of the collagen matrix can be challenging. To improve the ease of insertion, a specialized “goat” delivery clamp (Aesculap Chifa; B. Braun Melsungen, Nowy Tomyśl, Poland) was developed.^15^ However, there are still some inherent difficulties in using this instrument, and it may not be available to all. The aim of this Technical Note is to describe a surgical technique of matrix insertion by sliding the matrix down sutures during arthroscopic matrix-based meniscus repair.

## Surgical Technique

### Preoperative Patient Positioning

The surgical procedure is performed with the patient under general anesthesia. The authors’ preference is to perform the surgery with the leg supported on the table using a lateral support and foot rest. A tourniquet is placed over the operated leg at the level of the lateral support. The lower limb is cleaned and draped with the foot in a sterile stockinette.

### Meniscus Assessment and Preparation

**Fig 1:**
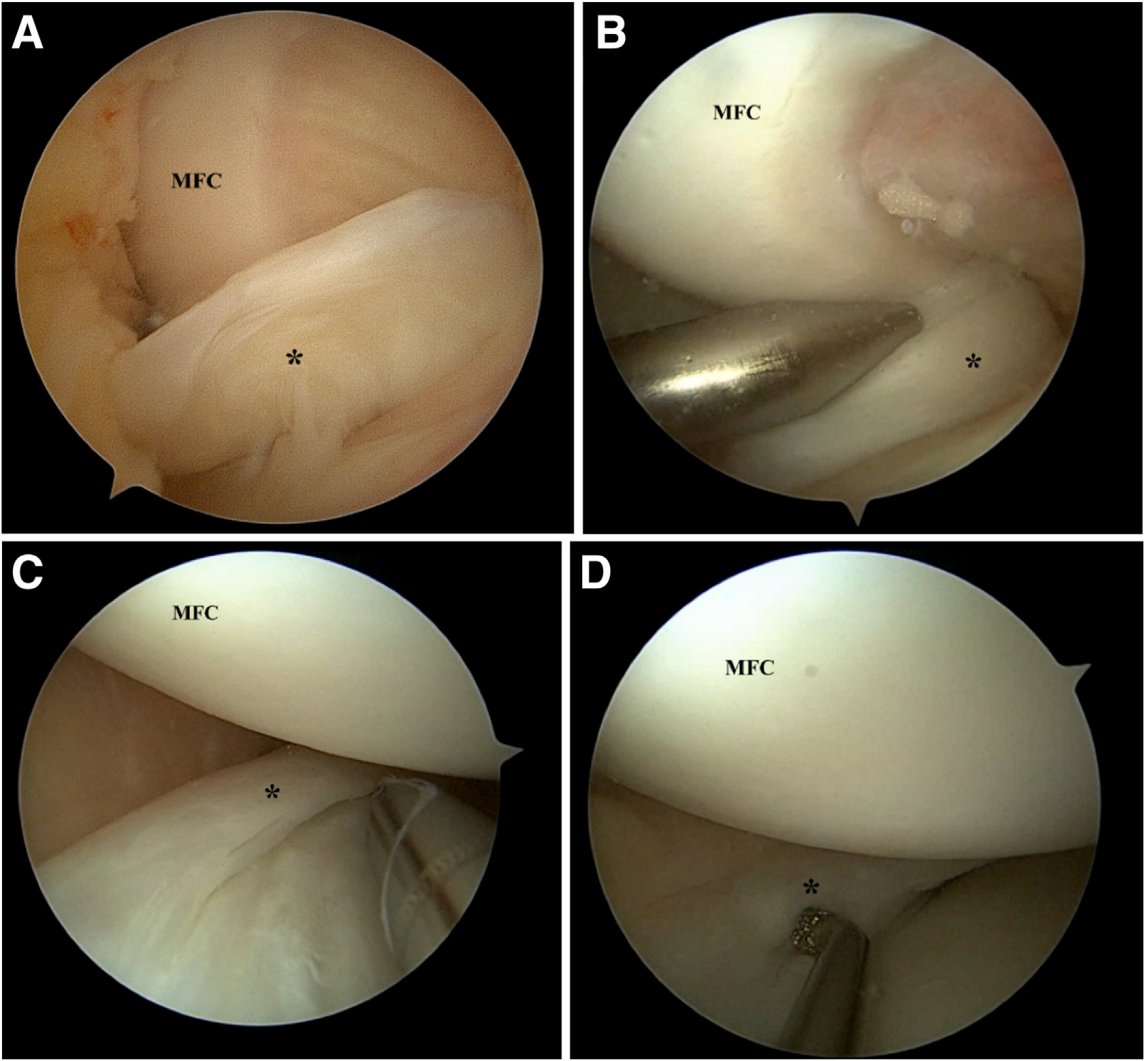
Left knee. Supine position with knee in valgus stress. Viewing from the anterolateral portal: (A) Medial meniscus bucket handle tear (∗) is visualized. (B) The bucket handle tear is reduced with a blunt obturator with the knee in semi-extension. Meniscus edges are abraded with a shaver (C) and rasp (D). (MFC, medial femoral condyle.)

Standard anterolateral and anteromedial portals are used (Video 1). A horizontal incision for the anteromedial portal is used to aid in maneuverability during meniscus repair. The menisci are assessed and a probe is used to determine the geometry of the meniscus tear. The bucket-handle tear of the medial meniscus is reduced with the knee in extension and valgus, using a probe or obturator to reduce the meniscus. For medial meniscus tears, percutaneous pie-crusting of the medial collateral ligament can be performed to improve visualization of the medial meniscus if the joint space is too tight. A meniscus rasp is then used to abrade the synovium and meniscus tear edges to improve healing (Fig 1).

### Meniscus Repair

**Fig 2:**
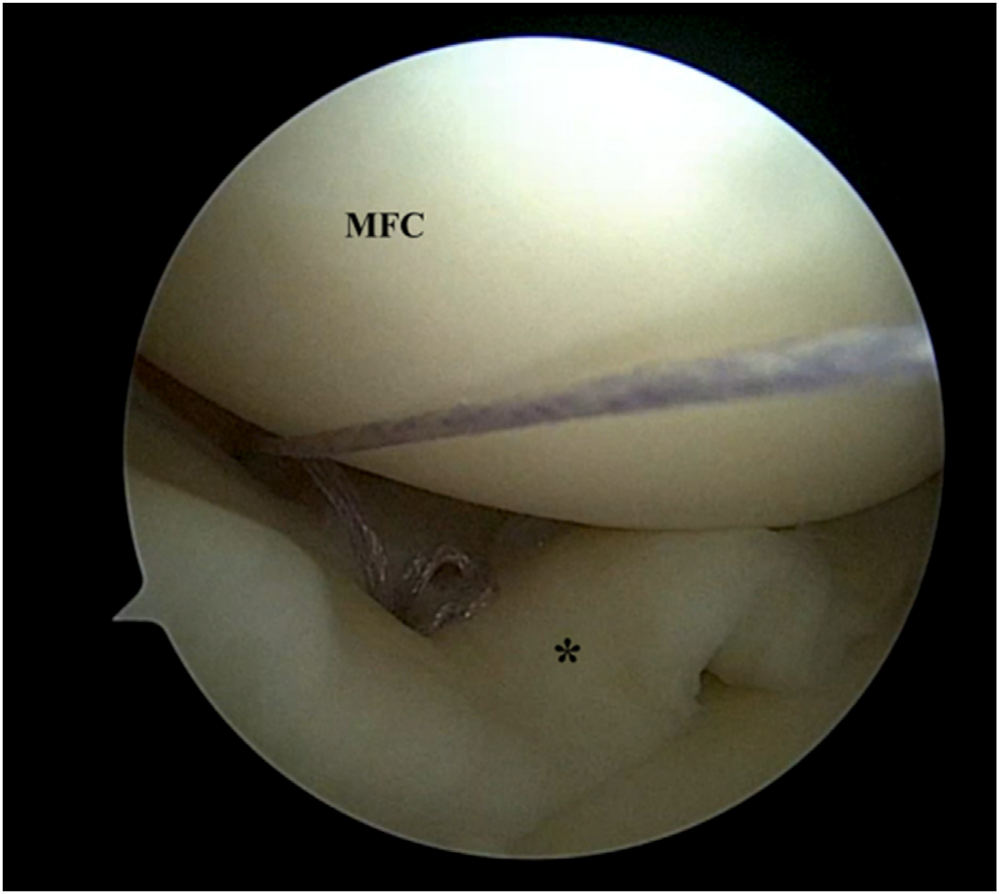
Left knee. Supine position with knee in valgus stress. Viewing from the anteromedial portal: Initial all-inside suture device is used to hold the reduction of the bucket-handle medial meniscus tear (∗).

**Fig 3:**
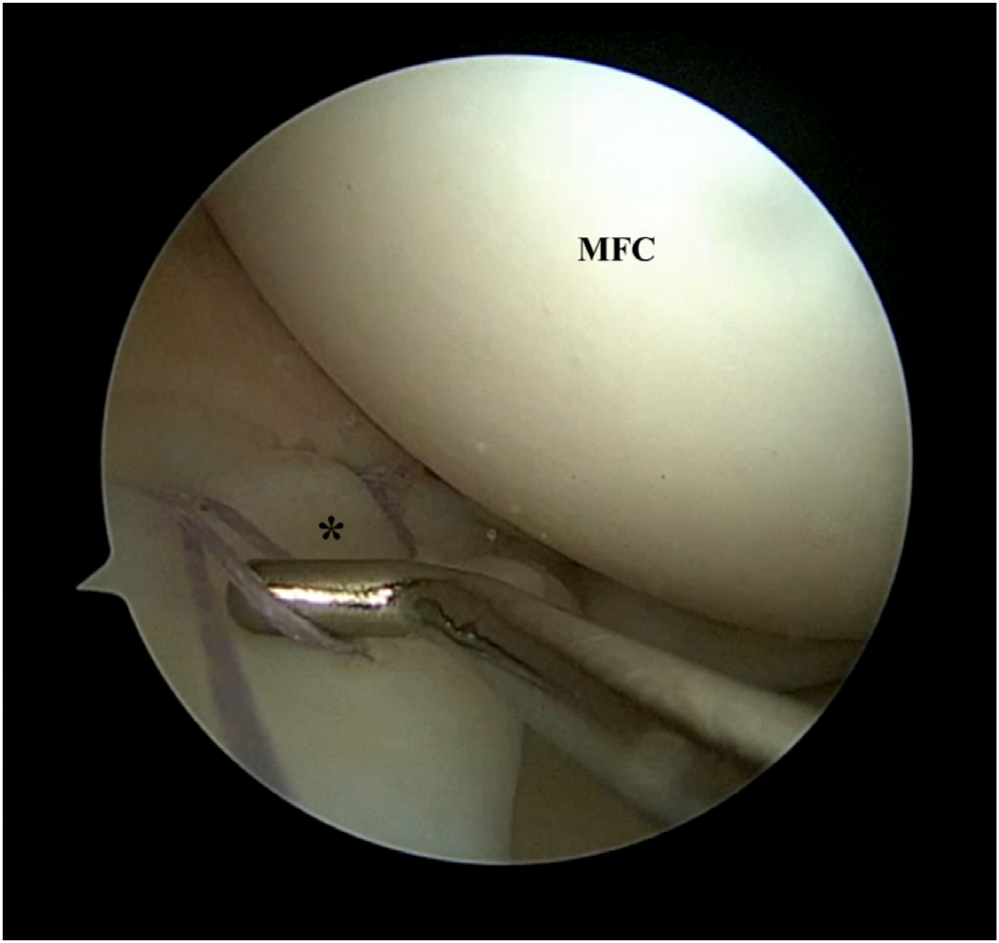
Left knee. Supine position with knee in valgus stress. Viewing from the anteromedial portal: Further all-inside suture devices are used to stabilize the meniscal tear (∗) adequately. (MFC, medial femoral condyle.)

**Fig 4:**
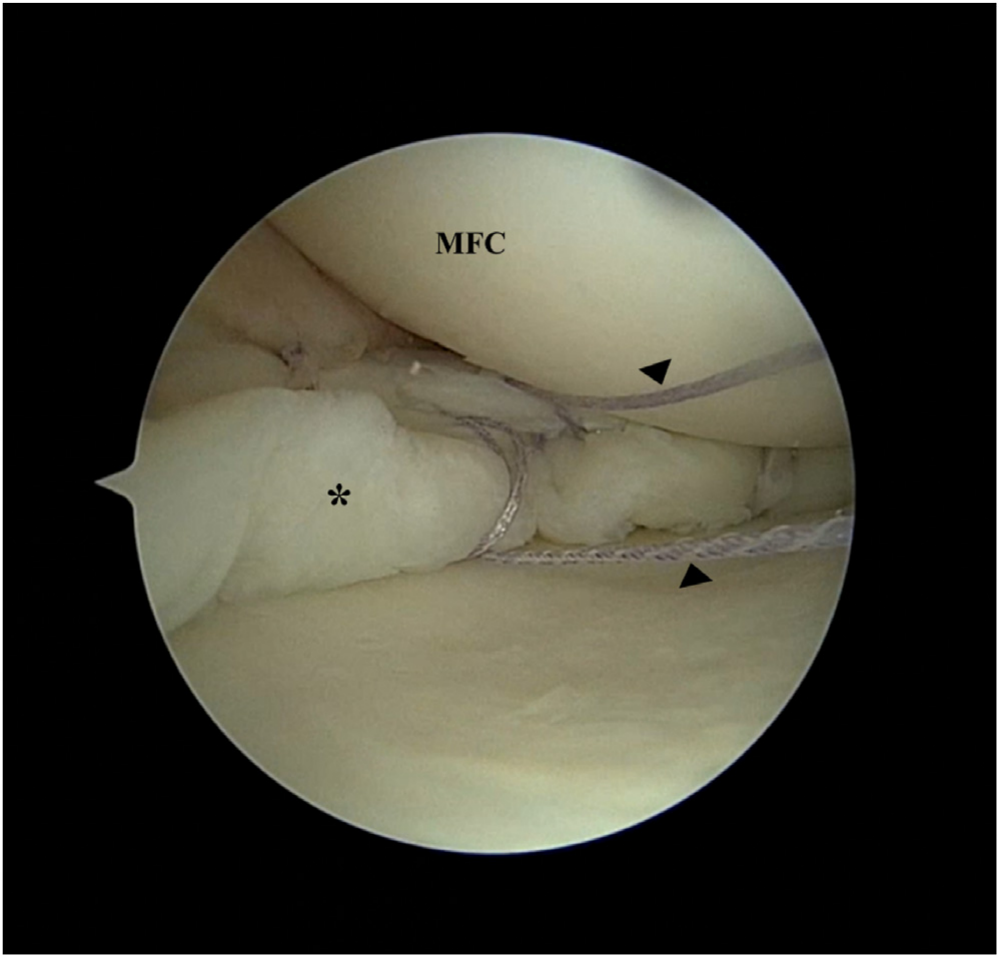
Left knee. Supine position with knee in valgus stress. Viewing from the anteromedial portal: At the area of interest of placement of the collagen matrix, the suture device usage is planned, with 1 all-inside suture device (arrowhead) placed above and 1 all-inside suture device (arrowhead) placed below the meniscus (∗). The sutures are not cut. (MFC, medial femoral condyle.)

Once the meniscus reduction is satisfactory, an initial all-inside repair device (TRUESPAN; DePuy Mitek, Raynham, MA) is used at the body-posterior horn junction to maintain reduction (Fig 2). Thereafter, several more all-inside devices are used to repair the meniscal body (Fig 3). At the area which is designated for collagen matrix application, the all-inside devices are used such that alternating sutures are situated above and below the meniscus after final tightening (Fig 4). These sutures are not cut immediately but are retrieved through the viewing portal to avoid interfering with subsequent repair stitches.

### Measurement of the Target Meniscus Area

**Fig 5:**
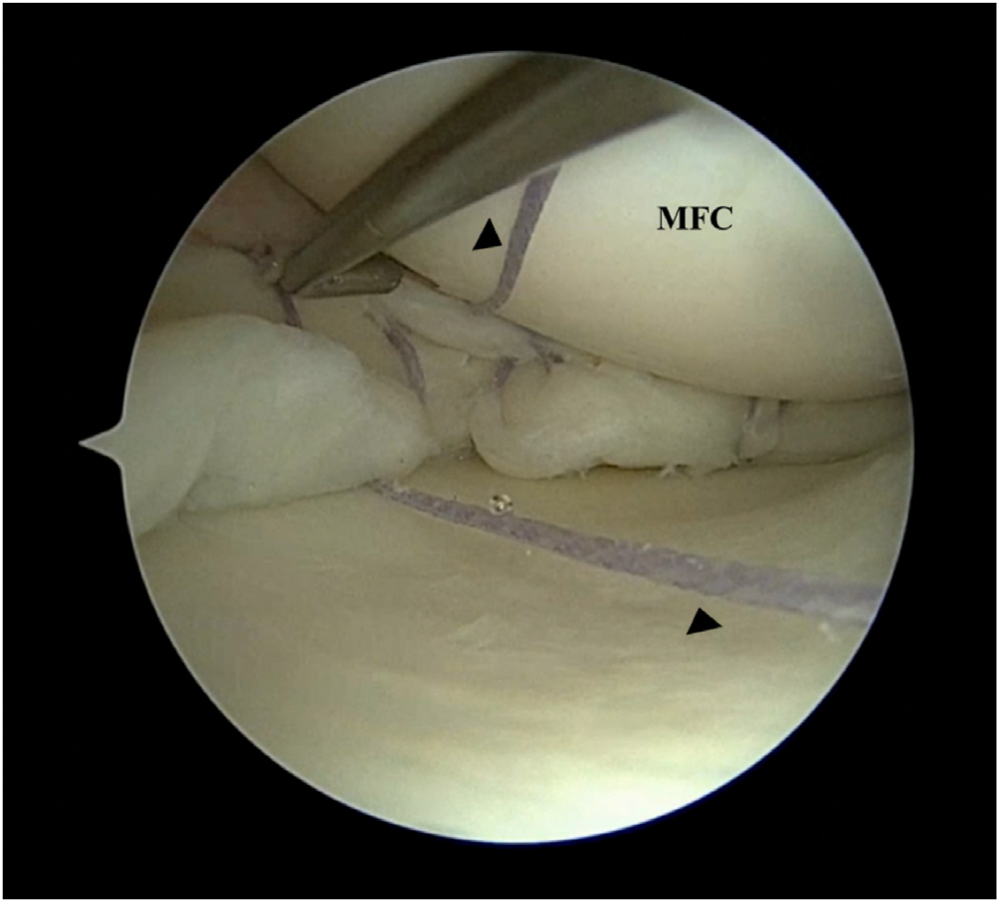
Left knee. Supine position with knee in valgus stress. Viewing from the anteromedial portal: A probe is used to measure distance between the sutures (arrowheads) so as to determine the location at which the sutures should be threaded through the matrix. (MFC, medial femoral condyle.)

The target meniscus area is measured using the arthroscopic probe to determine the length of collagen matrix needed. In addition, measurements are made between the sutures so as to determine the location at which the sutures should be thread through the matrix (Fig 5).

### Preparation of the Collagen Matrix

**Fig 6:**
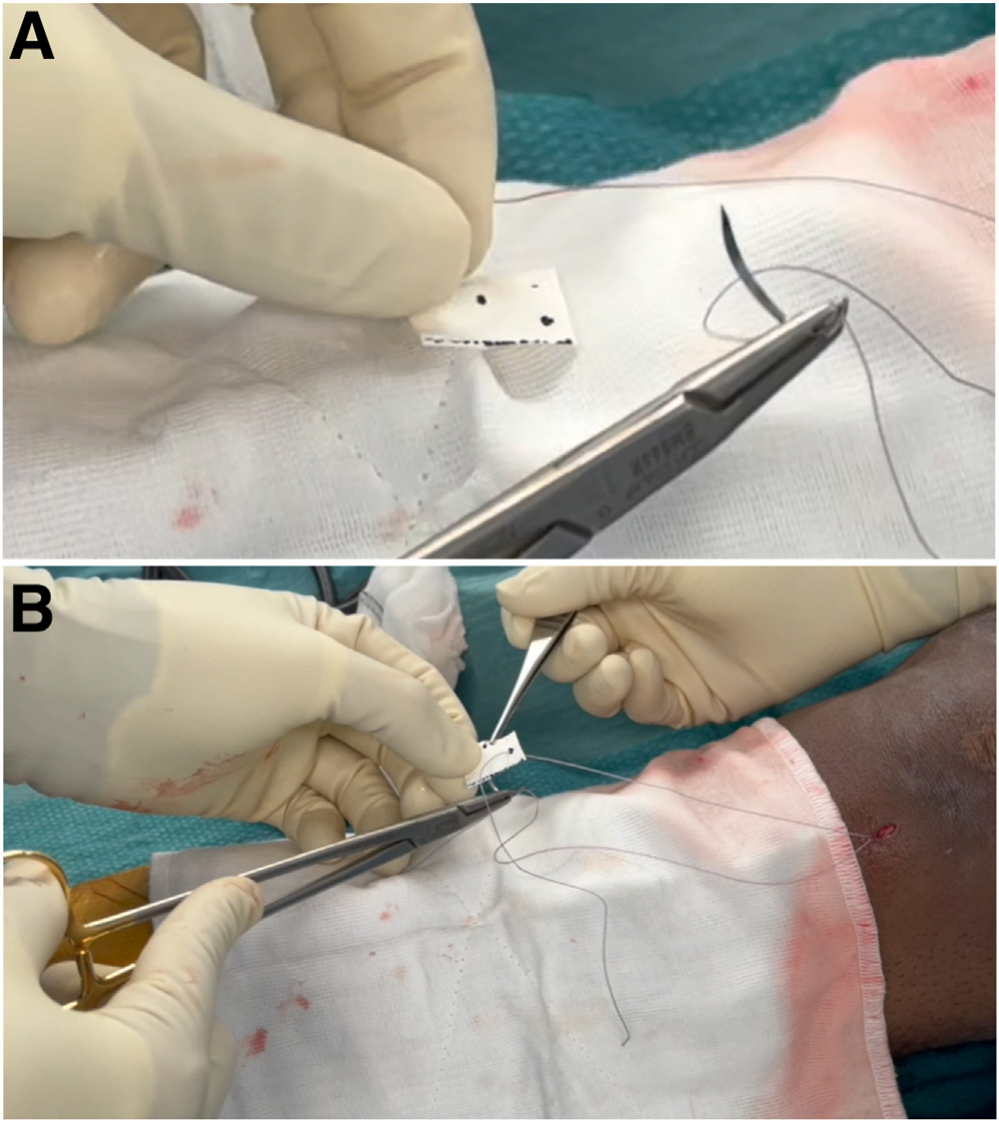
Matrix preparation: Collagen matrix is prepared ex vivo. (A) The matrix is measured, size trimmed appropriately, and distance between the sutures is marked. (B) The passing sutures are passed through marked areas using a trochar needle.

The collagen matrix (Chondro-Gide; Geistlich Biomaterials, Wolhausen, Switzerland) is prepared ex vivo. The smooth surface is marked with a surgical marker as the roughened surface is designed to face the meniscus. The matrix is measured and the size is trimmed appropriately. The distance between the sutures is marked, and the sutures are passed through the matrix using a trochar needle (Fig 6).

### Sliding Technique to Deliver the Collagen Matrix

**Fig 7:**
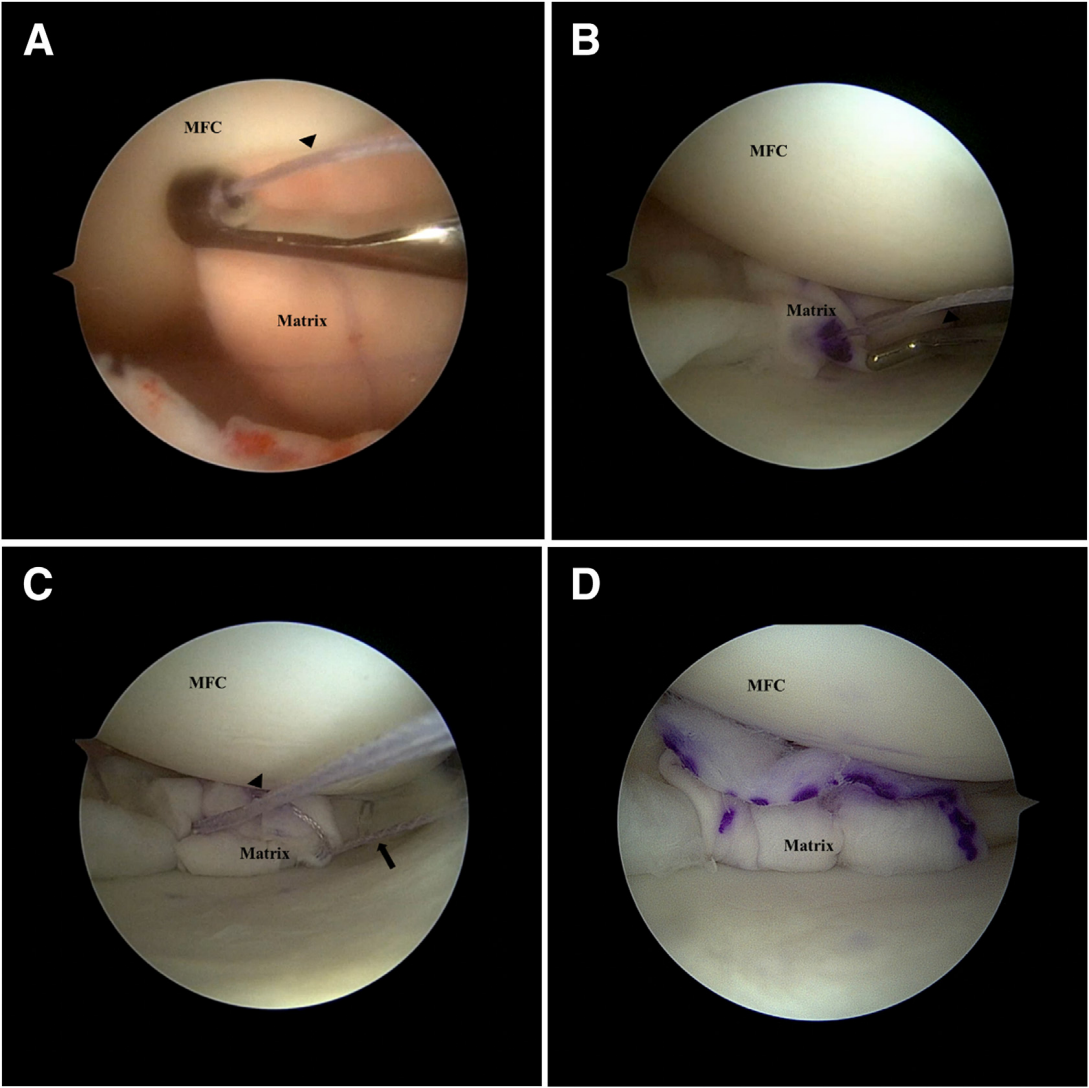
Left knee. Supine position with knee in valgus stress. Viewing from the anteromedial portal: The matrix smooth surface is marked with a surgical marker as the roughened surface is designed to face meniscus. (A) The matrix is slid down the passing sutures (arrowheads) onto the meniscal surface using a knot pusher. Note that the surgical marking is facing the cartilage surface. (B) Once the matrix is delivered on to the meniscus, the passing sutures are then tied over the matrix using a knot pusher for secure fixation. (C) Additional all-inside suture devices (block arrow) can then be used to improve fixation if needed. (D) Matrix is shown in its secure final position. (MFC, medial femoral condyle.)

Using the sutures that have been passed through the collagen matrix, the matrix is slid down along the sutures onto the meniscal surface using a knot pusher (DePuy Mitek, Raynham, MA). If more than 1 suture pair is used, the risk of the matrix “turning turtle” is reduced. If only 1 pair is used, it is important to ensure that the surgical marking is facing the cartilage surface (Table 1). Once the matrix is down on the meniscus, the sutures are tied over the matrix using the knot pusher. This achieves a secure fixation. Thereafter, additional all-inside repair devices can be used to improve fixation if needed (Fig 7).

**Table 1:** Pearls and Pitfalls

Pearls
A knee cannula can be used when dealing with multiple sutures so as to prevent soft-tissue bridges. Accurate measurement of the distance between the suture strands should be performed, and these measurements should be marked onto the matrix so as to ensure that the sutures do not cause the matrix to bunch up or tighten excessively once applied onto the meniscal surface. Tying of knots can be pushed toward the inferior aspect of the meniscus to prevent excessive knot profile.
Pitfalls
If only one pair of sutures is used, there is a risk of the matrix flipping in situ during delivery into the joint. Surgeons should mark the appropriate surface of the matrix so as to ensure they place the correct surface facing the cartilage. Inadequate suture management may result in entanglement and soft-tissue bridges that may be difficult to resolve.

### BMAC Injection

**Fig 8:**
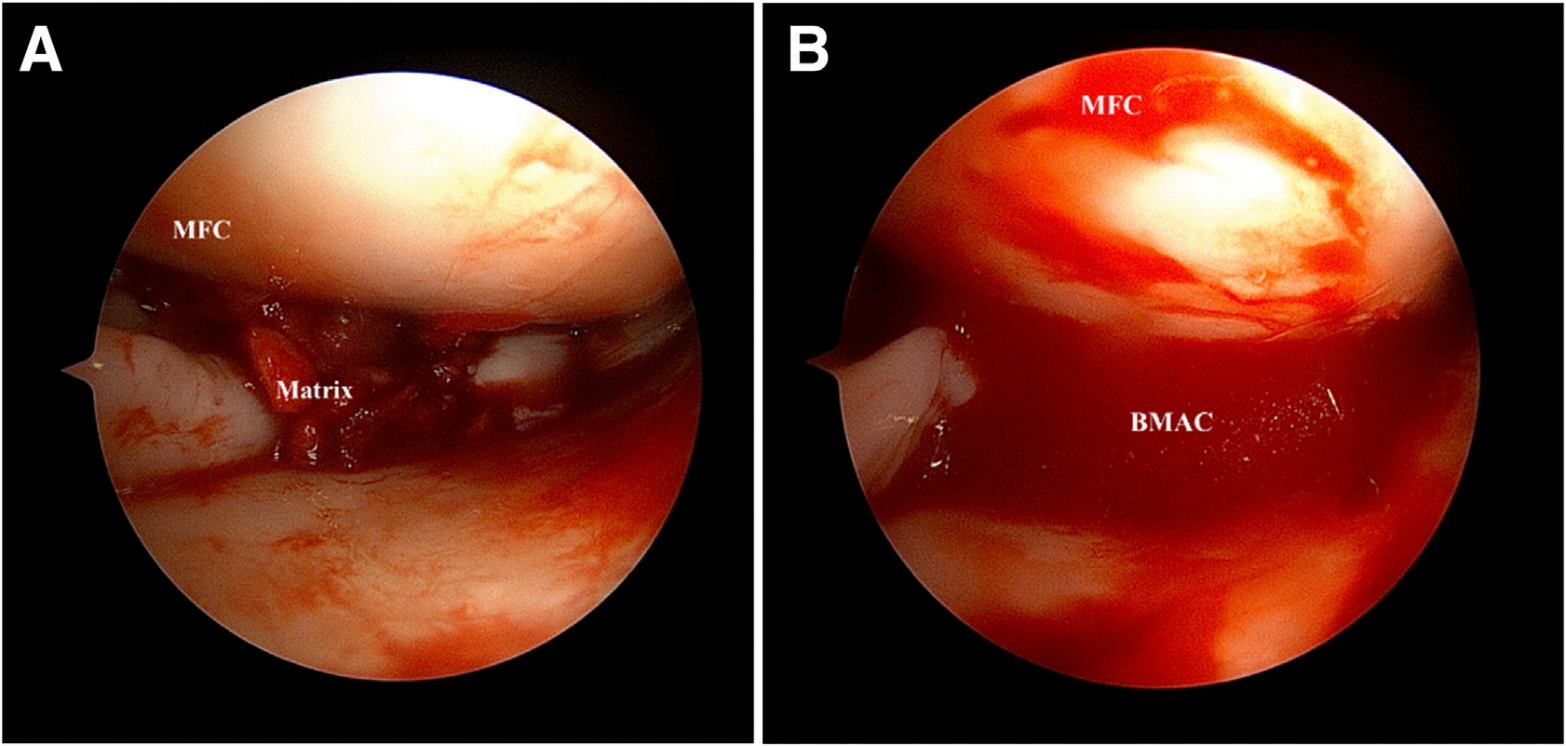
Left knee. Supine position with knee in valgus stress. Viewing from the anteromedial portal: (A) Fluid is drained out of the joint and dry arthroscopy is performed. (B) A spinal needle is inserted into the area of the matrix and previously harvested bone marrow aspirate concentrate (BMAC) is injected onto the matrix and peri-capsular region. (MFC, medial femoral condyle.)

Fluid is drained out of the joint and under dry arthroscopy or gas arthroscopy, a spinal needle is inserted into the area of the matrix. The previously obtained BMAC (Zimmer Biomet, Winterthur, Switzerland) can now be injected onto the matrix as well as the pericapsular region. The joint is not flushed again and closure of the portals can be performed (Fig 8).

### Postoperative Protocol

The operated lower extremity is placed in a knee brace. Range of motion is limited from 0 to 90° for 6 weeks. The patient is not allowed to weight bear for 6 weeks. Thereafter, range of motion exercises and full weight-bearing is allowed. A progressive strengthening program is then implemented.

## Discussion

There is a growing shift toward aggressive meniscal preservation.^1^, ^2^, ^3^ Surgical repair techniques continue to evolve for challenging tear patterns. The popularity of biological adjuncts has also grown in tandem in attempt to improve healing rates. Vascularity is a well-established prognostic factor for successful meniscus healing postrepair. In addition to vascularity, it is believed that improving the biological healing environment will also improve meniscal healing.^4^^,^^8^, ^9^, ^10^^,^ Orthobiologics such as BMAC have shown some positive results in improving short-term outcomes when combined with meniscus repair, as compared with isolated meniscus repair alone.^9^^,^^16^

Cartilage regeneration techniques often involve using scaffolds combined with BMAC. This delivery system has also been used for meniscus tears, given the belief that the scaffold can retain the BMAC within the area of interest. Piontek et al.^12^ described the arthroscopic matrix-based meniscus repair technique of using a collagen matrix (Chondro-Gide) in conjunction with BMAC. Five-year follow-up results of this technique showed statistically significant improvements in subjective scores and clinical assessment between preoperative, 2-year, and 5-year follow-up time points, with an overall survival rate at final follow-up of 88%.^13^^,^^14^

Accurate placement of the collagen matrix over the meniscus is critical. Although the collagen matrix is often easily handled during fluid arthroscopy, it can be challenging to secure it over the area of interest. A “goat” delivery clamp has been described, which involves placement of the matrix ex vivo into an expandable clamp that allows for ease of access and placement.^15^ Nonetheless, although using the “goat” delivery clamp can help, this equipment may not be available to all surgeons and requires a degree of familiarity to use proficiently. Our surgical technique of sliding the matrix down existing sutures is straightforward and easy. (Table 2). The technique allows for accurate placement of the matrix over the area of interest and is familiar to surgeons who are used to arthroscopic knot tying. This method ensures that the collagen matrix will be retained at the target meniscal area. Final stabilization of the collagen matrix can then be performed with additional all inside sutures as required. BMAC can subsequently be injected over the area under dry arthroscopy. The suture sliding technique for arthroscopic matrix-based meniscus repair using a collagen matrix is an easy and straight-forward surgical technique that allows for controlled stabilization of the collagen matrix over the target meniscal area.

**Table 2:** Advantages and Disadvantages of Our Technique

Advantages
This technique allows for an easy insertion of the collagen matrix without requiring additional equipment. No additional implant is required, as the sutures used to slide the matrix down were all-inside repair sutures that were previously used for meniscal repair. This method prevents erroneous placement of the matrix, as it will dock at the correct position due to the sutures passed through it.
Disadvantages
Cannot be used if there is no meniscal repair required before placement of the matrix (e.g., pure degenerative meniscus lesions that do not require repair). Multiple sutures may make suture management challenging.

## Disclosures

The authors declare the following financial interests/personal relationships which may be considered as potential competing interests: S.W.L.H. reports speaking and lecture fees from 10.13039/100009026Smith & Nephew and DePuy Synthes Mitek Sports Medicine. All other authors (T.Z.X.T., W.Z., J.T., L.T.J.T., K.T.L.) declare that they have no known competing financial interests or personal relationships that could have appeared to influence the work reported in this paper.

## Supplementary Data

Video 1 Knee arthroscopy is performed using the standard anteromedial and anterolateral portals. An acute bucket-handle tear of the medial meniscus is visualized. In preparation for meniscus repair, a meniscal rasp is used to abrade the torn edges, followed by a shaver, which is used to freshen the edges further. Medial meniscus reduction is then achieved by using a blunt trochar for reduction, while the knee is kept in semiextension and valgus. After reduction, an initial all-inside suture device is used at the junction of the body and posterior horn to maintain the reduction of the bucket handle tear. Additional all-inside sutures can then be used for further reinforcement of the meniscal body. An adequate number of all inside suture devices should be used to achieve good stability of the meniscus. At the area of interest for placement of the collagen matrix, it is important to plan the placement of the all inside sutures. Once these all-inside sutures are placed, they are tightened and the remnant suture is preserved and not cut. It is favorable to place each of the sutures on the superior and inferior aspect of the meniscus, in a vertical orientation, as this allows for optimal fixation of the collagen matrix thereafter. A probe is used to measure the distance between the sutures. This distance is used to appropriately size the collagen matrix. Both sutures are retrieved through the anterolateral portal using a suture retriever to prevent any soft-tissue interposition. The collagen matrix is marked ex vivo with the previously measured distance between the pair of sutures. The appropriate sutures are then passed through the matrix using a trocar needle. It is important to ensure that the superior suture goes through the superior marking on the collagen matrix to prevent entanglement. The matrix can now be slid down the sutures using a knot pusher. In this case, one suture pair is used. It is important ensure that the correct side of the matrix is facing the cartilage. This is done by making sure that the surgical marking on the matrix is visible. Once the matrix is in place, the guide sutures are tied and cut. Additional pairs of all-inside sutures are used to improve the fixation of the matrix. The matrix is now secured at the area of interest. Under dry arthroscopy, the previously harvested bone marrow aspirate concentrate is introduced over the matrix.
